# Perceived Benefits and Barriers to Chinese COVID-19 Vaccine Uptake Among Young Adults in China

**DOI:** 10.3389/fpubh.2022.825874

**Published:** 2022-06-03

**Authors:** Wei Luo, Siyu Song

**Affiliations:** School of Journalism and Communication, Xiamen University, Xiamen, China

**Keywords:** COVID-19 vaccination, Chinese young adults, health belief model, Chinese culture, traditional Chinese medicine, social benefits, trust in the government

## Abstract

Survey-based research has provided us with breadth regarding perceived benefits and barriers to COVID-19 vaccination among Chinese people. Most such research has been conducted within hypothetical COVID-19 vaccine contexts, and few studies are specific to young adults aged 18–40, a pivotal target population for COVID-19 vaccination. Now that the Sinopharm and Sinovac COVID-19 vaccines have been conditionally approved in China, qualitative investigation of young adults' perceptions of benefits and barriers to taking them is warranted. Such research may suggest potential candidate themes in the COVID-19 vaccination promotional messages targeting this population. Through in-depth interviews with 55 Chinese young adults and thematic analysis guided by the health belief model, social benefits and worry reduction emerged as significant positive factors in young adults' intention to vaccinate. Several novel barriers emerged as well, including perceptions that the vaccines' advantages are weak relative to non-medical preventions and beliefs regarding *Ti Zhi* (the individual human constitution), which confused some participants about their suitability for vaccination. The study also identified two modifying factors, trust in the government and perceived vaccine information insufficiency, both of which appeared to be indirectly associated with vaccination intention by augmenting the perceived barriers. The results suggest that more attention could be paid to young adults' cultural background when developing relevant health communications.

## Introduction

Vaccination is considered an important public health program component because of its effectiveness in inhibiting outbreaks and prevalence of infectious diseases (1). Currently, COVID-19 vaccines are being developed at an unprecedented speed (2). Access to safe and effective vaccines is critical to combat the still-worsening pandemic (3). As of October 12, 2021, over 300 COVID-19 vaccine candidates were under development worldwide (4).

The China National Medical Products Administration conditionally approved the Sinopharm and Sinovac COVID-19 vaccines (hereafter Chinese COVID-19 vaccines) on December 30, 2020, and February 5, 2021, respectively (5, 6), both of which are inactivated COVID-19 vaccines manufactured by Chinese companies. Adults aged 18–59 without contraindications were all eligible to get these vaccines (7). The Chinese publics often call them “Chinese vaccines” to distinguish them from those imported from other countries, such as Pfizer and AstraZeneca vaccines. Accordingly, during the study they were collectively termed “Chinese COVID-19 vaccines” and discussed jointly. Information on Chinese people's perceptions of COVID-19 vaccination in its initial phase is a prerequisite for designing effective promotion campaigns. However, little is known about such perceptions.

The World Health Organization has identified young people as a priority target population in the COVID-19 pandemic and warned that young adults aged 20–40 are increasingly driving the spread of COVID-19 virus in many countries (8, 9). Jobs and campus life are common reasons why young adults are vulnerable to COVID-19 infection (10). Several studies in China have shown that young participants under the age of 40 or 45 are less willing to receive COVID-19 vaccination than their older counterparts (11, 12). A recent survey conducted in China also reports that the vaccination rate in participants under 40 was lower than that of participants over 40 (13). For these reasons among others, Chinese young adults are an important target population for COVID-19 vaccination. Targeting health beliefs about COVID-19 vaccination constitutes an evidence-based approach for designing COVID-19 vaccine campaigns (14). To that end, this study aims to identify the beliefs this population holds.

The health belief model (HBM) has origins in preventive health behavior studies (15) and is a widely adopted conceptual framework underpinning much health promotion work (14). According to HBM, people's health beliefs play a determinant role in their health behavior (16). The term “health beliefs” refers to people's perceptions of health problems and behaviors. HBM predicts that when people perceive a health problem as a real threat and when the benefits of a recommended health behavior to address the problem outweigh its negative aspects (its “barriers”), they will engage in the recommended behavior (16). Accordingly, understanding a group's health beliefs is crucial for developing health messages that will promote those resonating beliefs on the desired behavior (14, 17). As benefits and barriers are the strongest predictors of health behavior (18), including in the context of Chinese people's COVID-19 vaccination intentions (12, 19–21), benefits and barriers are the entry points for this research effort.

Before and after the approval of Chinese COVID-19 vaccines, several HBM-guided, survey-based studies explored Chinese people's health beliefs regarding COVID-19 vaccinations. Items measuring perceptions of benefits include self-protection (19, 21, 22), protecting family members (20), preventing the spread of COVID-19 (23), worrying less (12), and using masks less (22). Barriers most commonly include vaccine efficacy (including duration of protection) (20–22), safety concerns (12, 20–22), and infection due to vaccination (23, 24). In some cases, researchers did not explicate the reasons for choosing such items (12, 20). In other cases, they borrowed and adjusted the items from other vaccination studies (such as influenza vaccination) (21) to develop COVID-19 vaccination-specific instruments. However, these revisions were not based on suggestions from interviews or focus groups with the targeted populations, but instead on researchers' subjective opinions. Fishbein and Yzer suggest that health communication researchers and practitioners must understand the beliefs about a certain health behavior from the perspective of the population under consideration (25). The items in aforementioned studies may leave out certain promising beliefs that could be identified through a qualitative investigation.

Additionally, most such research was conducted before the Chinese COVID-19 vaccines were approved (12, 21–23), meaning that participants' judgments and perceptions were about the benefits of and barriers to imaginary, hypothetical COVID-19 vaccines. However, the findings from recent research suggest that COVID-19 vaccine products' specific features influence participants' perceptions and intentions regarding vaccination. For example, Yu et al. (22) indicate that a vaccine's formal scientific legitimacy, that is, completion of all clinical trials and approval from health authorities, is the most important factor in participants' willingness to take it. Moreover, research by Lin et al. (12) reveals that Chinese participants trust domestic vaccines more than imported ones. Therefore, compared to hypothetical ones, the real COVID-19 vaccines with specific features provide a context for Chinese people to make decisions and for researchers to explore the associations between beliefs and intentions.

Building on the aforementioned research on health beliefs and COVID-19 vaccination, the current study leveraged qualitative approaches to elicit beliefs about receiving Chinese COVID-19 vaccines among Chinese young adults. We aim to inductively elicit such perceptions using in-depth interviews and thematic analysis. This qualitative inquiry will benefit related literature for its added research contexts (i.e., the specific target population and vaccine products) and can be useful in generating the essential beliefs that allow for the development of viable message strategies to promote vaccination behavior (14, 17, 26). Additionally, the study findings can be used as a reference for future research that relies on COVID-19 vaccination beliefs. The study is then guided by the following research question: What specific benefits and barriers to taking the Sinopharm and Sinovac COVID-19 vaccines do Chinese young adults perceive?

## Methods

### Data Collection

In-depth interviews were conducted to address the research question. Between January and March 2021, the researchers recruited participants through their WeChat social media platforms, specifying the following criteria: (a) resident of mainland China, (b) 18–40 years old, (c) had heard about Sinopharm and/or Sinovac vaccines. Each participant was encouraged to recruit another participant with a vaccination intention different from their own. In accordance with the grounded theory concept of saturation (27), when new insights were no longer being yielded from new participants, the recruitment process was terminated. After providing their informed consent, participants gave basic demographic information, such as gender, age, and education, via an online questionnaire (Table 1).

**Table 1:** Demographic information on participants.

		**%**
**Gender**		
Male	28	51%
Female	27	49%
**Age**		
18–30	31	56%
31–40	24	44%
**Education**		
Associate college	5	9%
Bachelor's degree	27	49%
Master's/PhD degree	23	42%
**Medical science background**		
Previous medical science experience	4	7%
No previous medical science experience	51	93%
**Health professional background**		
Work or worked in medical or healthcare institutions	2	4%
Never worked in medical or healthcare institutions	53	96%
**Other**		
Healthcare workers in the family	20	36%
Healthcare workers among close friends	9	16%
Healthcare workers both in the family and among close friends	7	13%
No healthcare workers either in the family or among close friends	19	35%

In summary, 55 young adults from 21 provinces in mainland China participated in the interviews. Twenty-nine participants intended to take a Chinese COVID-19 vaccine, with 8 of them having completed at least one dose. The remaining 26 had no vaccination intention at the time of the interview. None of the participants and no members of their families had been infected with COVID-19 at the time of the interview. Individuals were recruited and participated on a voluntary basis.

The interview process was implemented as follows: after drawing up an agreed initial interview protocol, each of the two researchers carried out three interviews, and then they compared notes and discussed and revised the interview protocol based on the results. Having agreed upon the revised protocol, each of the researchers continued interviewing participants on an individual basis. Each semi-structured interview lasted between 15 and 50 mins. The interviews were conducted via telephone or WeChat voice calls depending on each participant's preference. At the beginning of the interviews, participants were asked about their views on the COVID-19 pandemic. They were also invited to talk freely about their knowledge of Chinese COVID-19 vaccines (such as manufacturers, efficacy, and safety) and their attitudes about what they knew. Then, they were asked to describe their specific perceptions of benefits and barriers to taking these vaccines. Finally, they were asked to describe their thinking process in deciding whether or not to take the vaccines. During each interview, the researcher also compiled additional notes and observations on the process in an interview memo.

The interviews were conducted in Chinese and recorded, then transcribed verbatim and analyzed. The researchers' interview memos were also included in the analysis. Selected sections of the transcripts were translated into English for use in this article. A translator with a master's degree in English served as a research assistant and checked the authors' Chinese-English translations for accuracy.

### Data Analysis

Braun and Clarke's thematic analysis method was applied (28), and Nvivo 12 qualitative data analysis software used for data processing. First, after a deliberative review of all interview memos and transcripts, the researcher's open-coded four interview transcripts together, two randomly selected from participants who intended to vaccinate and two from those who had no vaccination intention, to verify dependability (29). Second, each researcher coded half of the remaining manuscripts to generate initial codes. These codes and associated data were then distributed among themes related to benefits, barriers, and decision-making processes. Researchers then jointly identified sub-themes related to different dimensions of benefits and barriers and other reasons for accepting or rejecting COVID-19 vaccines and distributed the initial codes among these subthemes. The inter-rater reliability measured by Cohen's Kappa coefficient was 0.87. The number of participants who supported each sub-theme was also calculated to reflect the prevalence of the sub-theme. Any disagreement between researchers was resolved through discussion.

## Results

Table 1 shows participants' demographic information. Participants were almost equally represented across gender with a mean age of 29 years, and the majority did not have a medical science (93%) or health professional background (96%). However, the majority of participants (65%) reported having healthcare workers in their networks of family or/and close friends.

Three types of benefits and five types of barriers emerged from thematic analysis. Not surprisingly, the benefit most reported by participants was self-protection (*n* = 38). Meanwhile, concerns about vaccine effectiveness (*n* = 38) and safety (*n* = 33) and the inconvenience of vaccinations (*n* = 12) were reported; these are common barriers frequently found in vaccination research (30, 31). However, some novel benefits and barriers specific to Chinese COVID-19 vaccines in the context of the COVID-19 pandemic in China were identified, and we focus on these here.

Eight of the 29 participants who intended to be administered Chinese COVID-19 vaccines have had at least one dose. They reported similar perceived benefits to the other 21 participants who have not received vaccines but somewhat different perceptions in barriers, with only one of them having slight concerns regarding the vaccines' safety. However, 10 of the participants who have not been administered any vaccines reported mild to moderate safety concerns.

Additionally, the study identified two modifying factors that may be indirectly associated with vaccination intentions by expanding the perception of barriers (14, 16). Scrutinizing these novel beliefs and modifying factors may yield new insights for scholars and practitioners concerned with developing vaccine promotion materials that uniquely target Chinese young adults.

### Benefits

#### Social Benefits

Thirty-five participants considered taking COVID-19 vaccines to benefit people around them and even the entire society. Through vaccination, they could “shoulder the responsibility of protecting others” (Pan, female, 21). The “others” participants were most concerned about were their family members, especially older parents and young children. For example, Ren (male, 27) stated, “my parents and children will be fine because they stay indoors most of the time. But as young adults who work elsewhere, we could possibly take the virus home.” Therefore, by getting vaccinated, “you wouldn't pass the virus on to people in contact with you, especially your family” (Fei, male, 40). Furthermore, participants also believed that vaccination could prevent them from “messing up other people's lives” (Wang, male, 28). In China, contact with a COVID-19 infected person not only exposes one to infection, but also requires mandatory quarantine for at least 14 days in accordance with epidemic prevention policies. Accordingly, participants thought that being vaccinated could minimize the possibility of people in contact with them facing such measures because they would be less likely to contract the virus.

Some participants also recognized the potential for broader social benefits that extended beyond protecting others. They saw themselves as intermediaries in COVID-19 transmission. Therefore, the vaccination could “cut off the spread of the virus” (Ge, female, 32) and “protect the people around me as well as those around them. So, the ratios of people who are safe would geometrically increase” (Le, female, 31). Based on such perceptions, Chen (female, 26) concluded, “Vaccination is one for all and all for one. If everybody got vaccinated, maybe finally COVID-19 could just be completely gone.”

#### Reducing Worry

A pivotal benefit (*n* = 27) was the perception that vaccination could “offer […] peace of mind” (Sun, male, 27) and reduce the “need to worry in the short term” (Zhu, male, 30). It was thought that “even if the vaccination turned out to be not effective, at least it would be useful psychologically” (Zhu). This psychological effect enabled those young adults to “face work and life in a more confident way” (Chu, male, 35).

In most cases, participants seemed less worried about risks to their physical health if they contracted COVID-19. As Chen (female, 26) expressed, “for us young people, the threat of this virus is not great. It's not an incurable disease.” Their true concerns were about the social consequences that becoming infected with COVID-19 could have for their families and their work. For example, participants who cared for children or aging parents were very worried, as expressed by Teng (female, 36): “I am the pillar of my family. If I accidentally get infected with COVID-19, the entire family will collapse.” Others had work-related worries, such as: “If I am infected and quarantined, my income will decrease” (Tang, female, 23) and “I'll have to impose on my colleagues to take over my job” (Sun, male, 27).

### Barriers

#### Weak Perceived Relative Advantages

Eighteen participants doubted the advantages of vaccination in preventing COVID-19 infection relative to non-medical prevention measures, especially mask wearing. One participant argued, “I don't think vaccines are more effective than mask wearing [in protecting us from infection]. Whether or not you need to wear a mask if you are vaccinated has not been verified. But wearing a mask and not being vaccinated has been proven effective” (Dong, female, 30). Another participant was concerned that after vaccination, “you won't be able to feel whether you are stronger or not, and you won't know if your body has successfully generated antibodies” (Zhao, female, 24). Perceived inability to observe the effects of vaccination lessened these participants' confidence in the vaccines' advantages. Accordingly, they regarded non-medical prevention (especially mask-wearing) as the primary and most effective protective measures, with vaccination as a kind of “assistance” that “made up for accidents” (Wang, male, 28), enabling people “to be better protected on the basis of mask wearing” (Ge, female, 32).

#### “Maybe My *Ti Zhi* Is Not Suitable”

One novel barrier that emerged was that some participants (*n* = 17) had great doubts about whether their *Ti Zhi* was suitable for COVID-19 vaccination. A comment that was typical of many came from Yang (female, 32), who said, “I believe Chinese COVID-19 vaccines are very safe, but maybe my *Ti Zhi* isn't suitable for taking them.” *Ti Zhi* here refers to a term in traditional Chinese medicine involving an individual person's overall constitution (32). It not only involves the individual's physical conditions and mental states but also carries cultural notions. Chinese people's opinions on *Ti Zhi* are usually connected with other traditional cultural conceptions such as *Yin, Yang, Qi, Xue*, and *Wuxing* (32).

For some participants, concerns about their *Ti Zhi* seem related to their physical conditions or previous medical experience. Zhou (male, 24) recalled his allergies to previous medications revealed by “scratch tests” and remarked, “I'm not sure whether I'm allergic to these vaccines as well.” Similarly, Ma (male, 25) was worried about his family's history of heart problems and explained, “I am afraid that vaccination will eat up too many of my nutrients or energy and even burden my heart.” For others, concerns about *Ti Zhi* were underpinned by a lack of confidence in their own immunity. Luo (female, 20) believed that her immune system had been weak since childhood and concluded that while “the vaccination could be fairly safe for others, if my *Ti Zhi* cannot restrain the inactive virus, it could very well-turn me into an asymptomatic carrier (of COVID-19).”

However, for even more participants, *Ti Zhi* was only a vague and blurred perception of their physical condition. They could not explain in detail what their *Ti Zhi* really was but used it to validate their distinctiveness. Miao (female, 31) expressed the opinion that “everyone is structured differently. So because of prevalent *Ti Zhi* differences, it's fine for some people to be vaccinated but not suitable for me.” *Ti Zhi* differences were perceived to exist not only among Chinese people but also between Chinese people and the non-Chinese participants in the Chinese COVID-19 vaccine clinical trials. Yu (female, 33) argued, “most Sinopharm clinical trials were carried out abroad. So there are no statistics on the suitability of the vaccine for Chinese people. After all, the *Ti Zhi* of the foreigner is different from that of the Chinese.” Belief in *Ti Zhi* differences led some participants to conclusions like “COVID-19 vaccination is actually a wise choice for most people. But personally I've decided not to get vaccinated” (Xu, male, 32).

### Modifying Factors

#### Trust in the Government

Twenty-nine participants conveyed that their trust in the Chinese government reduced their concerns regarding the safety of Chinese COVID-19 vaccines, which represented a common barrier reported by other participants. This trust stemmed from multiple reasons. First, it came from the perceived reliable image presented by the Chinese government to the public, which made them think that the government “will not do risk-taking things” (Ren, male, 27). Shi (male, 26) explained the relationship between this image and his perception on vaccines: “The Chinese government has always prioritized the safety of its people. So I think since it recommend the public to get these vaccines, the vaccines must have been verified extremely strictly and would be safe.”

Second, the trust is based on the government's “free vaccination” policy. Le (female, 31) argued, “look, the government said the vaccines are free. But why? It is to encourage everyone to get vaccinated. If the officials are not confident that the vaccines are really safe, how dare they make such a decision?”

Third, some participants believed that the issue of COVID-19 vaccines “is not merely a public health thing, it has also involved the political field” (Ge, female, 32). Ge has always been less trusting of vaccines made in China, but she has no doubts about the safety of Chinese COVID-19 vaccines since these vaccines “are not only used in China, but also donated or sold to other countries.” Therefore, “once these vaccines have safety problems, it will have a very negative impact on the country's image and international reputation” (Ge). Based on these judgments, she believed the Chinese government “will not let the vaccines have safety bugs” (Ge).

#### Vaccine Information Insufficiency

The perception that there was a lack of information regarding Chinese COVID-19 vaccines disclosed by the government influenced some participants' (*n* = 16) perceptions of safety. It was perceived that the government likely “released part of the information while omitting the rest” (Xu, male, 32). Participants complained that most of the information disclosed centered on “how to reserve a vaccine.” However, as Li (female, 28) observed, “the government delivered very limited information about the vaccine itself. There is nothing so far that explains the vaccine very clearly.” These participants expected “scientific statistics” provided by “a credible institution.” Xie (female, 27) compared Chinese COVID-19 vaccines with ordinary medicine and elaborated on the need for more detailed requirements for providing “scientific statistics” on side effects:

When you are taking or being injected with a certain kind of medication, the instruction booklet tells you which kinds of patients it is and is not suitable for. Side effects and their frequency will also be detailed. However, up to now, I've found very little such information on Chinese COVID-19 vaccines, and I'm not sure whether I will have access to instructions like that if I take the vaccine.

Absence of statistical data on side effects made participants like Xie feel “less secure” about the vaccines and thus refuse to be vaccinated. Some participants, although willing to take the vaccine, emphasized that they “will keep waiting and observing to see if more detailed information about the vaccine will be disclosed or not” (Han, male, 30) before making a final decision.

A few participants found it suspicious that no news on Chinese COVID-19 vaccine side effects was being reported. “To date, a fairly large number of people have been vaccinated. Allergic reactions cannot be completely avoided no matter how secure the vaccines are,” Wang (male, 28) explained. Meanwhile, sizeable media coverage of the severe side effects of vaccines manufactured by companies in other countries added weight to their doubts. “When you look at our country's media reports on accidents and problems with vaccines abroad, you have to wonder how they can claim that our vaccines have few problems. I don't think this makes sense” (Miao, female, 31). Chang (male, 33) interpreted the media coverage similarly and assumed that “Chinese COVID-19 vaccines may lead to severe side effects, but these are not covered by the media.” On this basis, they concluded that vaccines hold “potential threats, [and] it is better not to take them” (Miao, female, 31).

## Discussion

This study intends to fill a specific gap in the literature on COVID-19 vaccination behavior research by addressing the scarcity of in-depth knowledge on the benefits of and barriers to taking Chinese COVID-19 vaccines as perceived by Chinese young adults within the context of the real (vs. hypothetical) vaccines. This qualitative research may alert government policymakers and other stakeholders to the perspectives held by this key population group (33). The benefits and barriers identified from analysis of participants' narratives suggest potential candidate themes for more tailored messaging. The commonly perceived benefits (i.e., self-protection) and barriers (i.e., safety, efficacy, and practicality issues) frequently identified in research into COVID-19 and other vaccines could remain key targets of Chinese COVID-19 vaccine promotion campaigns. Focusing on the novel benefits and barriers that emerged in the present study may strengthen such campaigns. Nevertheless, additional research, such as experimental studies concerning the effects of different messages and/or statistical examination regarding the associations between these novel beliefs and vaccination intentions, is still needed.

In terms of social benefits, participants stressed the positive effects their own vaccination would have for the others around them and the wider community. Essentially, these perceptions indicate young adults' altruism and prosociality, which are qualities that can be appealed to in order to strengthen their COVID-19 vaccination intention (34, 35). In this study, participating young adults habitually focused on social benefits in their decision-making process. Their inheritance of a collectivistic culture may be a reason for this preference (36). Böhm et al. (37) find that prosocially motivated vaccination among healthcare personnel was more likely to occur in South Korea, another country with a collectivistic cultural background, than in the US, a more individualistic culture. The majority of Chinese young adults fall into the group with collectivistic cultures. Accordingly, the social benefits of getting vaccinated reflect one potential belief that can be highlighted in vaccination promotion messages among Chinese young adults.

Relatedly, reducing worry is a perceived psychological benefit that motivated Chinese young adults to get COVID-19 vaccinations. Worry about infectious diseases has been corroborated as strongly associated with the adoption of protective behaviors (38), and this finding may partly interpret the mechanism behind such relationship. Notably, for most participating young adults, it was not the potential effects of a COVID-19 infection on their own health that caused their worries but rather their concerns about the consequences of such an infection for their families and work. One possible explanation is that Chinese cultural teaching on within-family interdependence and prioritization of group goals and interests (e.g., in a work team) compels young adults to consider the negative impacts on others if they become infected (39–41). Accordingly, presenting vaccination as a solution to avoid such risks and reduce related worries could be considered as a candidate persuasive theme in future COVID-19 vaccination promotions.

At the same time, culture can sometimes impede health promotions. The cultural notion of *Ti Zhi* is a typical example. Notably, young adults “diagnosed” themselves as having a *Ti Zhi* unsuitable for COVID-19 vaccination, indicating a subjectively based perception of risks rather than a real hazard estimated by health professionals. Some participants based their assessment of their *Ti Zhi* on specific physical conditions. However, none of those participants had discussed their *Ti Zhi*-related concerns with a healthcare provider. Other participants whose views and perceptions of their *Ti Zhi* were rather vague and unclear used this culturally-based medical notion to rationalize their pessimism bias. On the basis of differences in *Ti Zhi*, they overemphasized the possibility of experiencing rare side effects. Dutta emphasizes that health interventions should be sensitive to the cultural contexts in which health experiences are situated (42). When the study was being conducted, we observed that Chinese COVID-19 vaccine promotion messages appeared to include very little information in a traditional Chinese medical context, such as the relationship between *Ti Zhi* and vaccination. Taking the promotion messages on the website of the China National Health Commission and the social media of *People's Daily* as examples, those messages mentioned the situations in which people should not be vaccinated, such as pregnancy and lactation, but did not mention traditional Chinese medicine (43, 44). Future promotional messages could address Chinese young adults' *Ti Zhi*-related doubts by incorporating traditional Chinese medicine information.

Another novel barrier reported by participants was the perceived vaccination's weak advantages relative to non-medical prevention measures. As an innovative therapeutic intervention for COVID-19, the effects of COVID-19 vaccination were unknown to those participants. Rogers argues that the perceived relative advantages of preventive innovations, such as vaccines, are inherently low because the benefits of adopting the innovation are often delayed and largely intangible (45). Persuasive messages could nevertheless be used to convey the relative advantages of COVID-19 vaccination.

The participants who had already completed at least one vaccine dose had less vaccine safety concerns than those who intended to vaccinate but have not yet performed the behavior. Given that vaccination behaviors may remain relevant even to vaccinated individuals as the COVID-19 situation and vaccine safety and effectiveness research still unfold, future research should dive deeper into the beliefs of vaccinated individuals vs. non-vaccinated ones.

Chen et al. report that fake vaccine concerns is one of elderly (aged 65 or above) Chinese adults' perceived barriers (46), which was not reported by the participating Chinese young adults in our study. This difference may be due to the dissimilarity in research contexts, rather than a difference between age groups. Specifically, Chen et al.'s research was done before Chinese COVID-19 vaccines were conditionally approved for use. Through referring to prior incidents of vaccine safety issues, their participants were convinced that fake vaccines would be manufactured by some companies for monetary gains. However, within the context of the available vaccines, our participants stated that the vaccines were developed with funding from the Chinese government, under an extremely strict supervision. Thus, “we can't get fake COVID-19 vaccines under this situation” (Yang, female, 32). Accordingly, vaccine products ought to be well-considered in the future COVID-19 vaccination research.

This study also finds two modifying factors that may influence young adults' perceptions of Chinese COVID-19 vaccines, especially regarding their safety, and thus indirectly impact vaccination intentions. The first factor is young adults' trust in the Chinese government. Trust in governments and public authorities have been associated with COVID-19 vaccination intentions and behaviors (47–50). The findings of the present study reveal a mechanism underlying this association. Participating young adults reported that trust in the government reduced their safety concerns regarding the vaccines recommended by the government and, accordingly, improved their vaccination intentions. Chen and his colleagues' research in Chinese elder adults' beliefs in COVID-19 vaccination also found an association between trust in the government and vaccination intentions (46). Participants in their study expressed that the trust enhanced their self-efficacy regarding vaccination, leading to intentions to get vaccinated. These different findings by age underscore the importance of investigating beliefs within older and younger subgroups of Chinese adults.

The second modifying factor is young adults' perceptions of insufficient vaccine-related information. Specifically, they were unsatisfied with the availability of risk information regarding Chinese COVID-19 vaccines. The absence of a detailed narrative and statistical information about the vaccine risks evoked participants' worries on safety issues, thereby decreasing their willingness to be vaccinated. However, the effects of risk information disclosure on vaccination behaviors are ambiguous. Some studies report positive relationships between provision of vaccine risk information and subsequent behavioral outcomes (51), while others observe negative associations (52, 53). Therefore, additional research is needed to examine the relationship between Chinese young adults' exposure to Chinese COVID-19 vaccine-related risk information and their vaccination intentions.

## Theoretical Contributions, Practical Implications, and Limitations

First, this research updates pre-approved and hypothetical COVID-19 vaccination research by setting the research in the context of actual and specific vaccine products to elicit the targeted population's health beliefs regarding vaccination. This added context makes the study a valuable supplement to previous research.

Second, this research bridges the gap between HBM theory, an individual cognition-based behavior change model, and cultural sensitivity approaches (54) by identifying perceived social benefits and the *Ti Zhi*-related barrier to vaccination, which are associated with Chinese culture. Several elements of Chinese young adults' cultural background, such as interdependence, collectivism, and Chinese traditional medicine, could be taken into account in designing effective, persuasive vaccination messages and remove culture-based barriers that are unlikely to be overcome by approaches based on individual rationality (55).

Our findings should be interpreted in light of several constraints. First, our study only captured snapshots of Chinese young adults' perceptions of the Sinopharm and Sinovac vaccines in their initial stages, and longitudinal tracking of perception changes is needed. These findings should not therefore be considered the final word on Chinese young people's perceptions of Chinese COVID-19 vaccines nor generalized to other populations or to perceptions of other COVID-19 vaccines. Second, our data was obtained from a convenience sample. A significant feature of WeChat, the social media platform used to recruit the study participants, is that the information posted by users can only be seen by their followers, who are mostly their acquaintances. Although we attempted to expand representation by encouraging participants to recruit others, participants were of relatively homogenous socioeconomic status. The study lacked samples from medically underserved populations, such as rural residents and people with less education and health care access. This poses limitations to the applicability of these findings. Surveying those populations will uncover a wider range of health beliefs that affect national uptake of Chinese COVID-19 vaccines. Last, most of the participants (*n* = 47) had never lived in COVID-19 outbreak areas, and neither they nor their family members had been infected with COVID-19. Young adults who have lived in outbreak areas, have been infected, or have significant others who have been infected may have different perceptions of vaccination. Additional research exploring their health beliefs is needed and should be compared with the present findings.

In conclusion, our study suggests that cultural and context-sensitive factors ought to be given particular consideration in future research and health communications regarding the COVID-19 vaccination.

## Data Availability Statement

The raw data supporting the conclusions of this article will be made available by the authors, without undue reservation.

## Ethics Statement

Ethical review and approval was not required for the study on human participants in accordance with the local legislation and institutional requirements. The patients/participants provided their written informed consent to participate in this study.

## Author Contributions

WL wrote the manuscript. WL and SS revised the manuscript. All authors contributed to the data collection and analysis.

## Conflict of Interest

The authors declare that the research was conducted in the absence of any commercial or financial relationships that could be construed as a potential conflict of interest.

## Publisher's Note

All claims expressed in this article are solely those of the authors and do not necessarily represent those of their affiliated organizations, or those of the publisher, the editors and the reviewers. Any product that may be evaluated in this article, or claim that may be made by its manufacturer, is not guaranteed or endorsed by the publisher.

## References

[1] World Health Organization. Vaccines and Immunization: What is Vaccination? (2020). Available online at: https://www.who.int/news-room/questions-and-answers/item/vaccines-and-immunization-what-is-vaccination https://www.who.int/news-room/questions-and-answers/item/vaccines-and-immunization-what-is-vaccination (accessed November 30, 2021).

[2] Graham B. Rapid COVID-19 vaccine development. Science. (2020) 368:945–6. 10.1126/science.abb8923 32385100

[3] World Health Organization. COVID-19 Vaccines. (2021). Available online at: https://www.who.int/emergencies/diseases/novel-coronavirus-2019/covid-19-vaccines (accessed November 3, 2021).

[4] World Health Organization. COVID-19 Vaccine Tracker and Landscape. (2021). Available online at: https://www.who.int/publications/m/item/draft-landscape-of-covid-19-candidate-vaccines (accessed October 12, 2021).

[5] China National Medical Products Administration. China National Medical Products Administration Conditionally Approved the Registration Application of Sinopharm COVID-19 Inactivated Vaccine (Vero cell). (2020). Available online at: https://www.nmpa.gov.cn/zhuanti/ypqxgg/gggzjzh/20201231193329157.html (accessed November 3, 2021).

[6] China National Medical Products Administration. China National Medical Products Administration Conditionally Approved the Registration Application of Sinovac COVID-19 Inactivated Vaccine (Vero cell). (2021). Available online at: https://www.nmpa.gov.cn/zhuanti/yqyjzxd/yqyjxd/20210206154636109.html (accessed November 3, 2021).

[7] National Health Commission. COVID-19 Vaccination Begins for Key Groups in Beijing. (2021). Available online at: http://en.nhc.gov.cn/2021-01/04/c_82636.htm (accessed March 5, 2022).

[8] World Health Organization. Young People and COVID-19: Behavioural Considerations for Promoting Safe Behaviours. (2021). Available online at: https://www.who.int/news/item/14-06-2021-behavioural-considerations-for-youth (accessed March 3, 2022).

[9] Wan W Balingit M. WHO Warns Young People are Emerging as Main Spreaders of the Coronavirus. (2020). Available online at: https://www.washingtonpost.com/health/who-warns-young-people-are-emerging-as-main-spreaders-of-the-coronavirus/2020/08/18/1822ee92-e18f-11ea-b69b-64f7b0477ed4_story.html (accessed March 3, 2022).

[10] Johns Hopkins Medicine. Coronavirus COVID-19: Younger Adults Are at Risk, Too. (2020). Available online at: https://www.hopkinsmedicine.org/health/conditions-and-diseases/coronavirus/coronavirus-and-covid-19-younger-adults-are-at-risk-too (accessed March 3, 2022).

[11] Wang C Han B Zhao T Liu H Liu B Chen L. Vaccination willingness, vaccine hesitancy, and estimated coverage at the first round of COVID-19 vaccination in China: a national cross-sectional study. Vaccine. (2021) 39:2833–42. 10.1016/j.vaccine.2021.04.020 33896661 PMC8043613

[12] Lin Y Hu Z Zhao Q Alias H Danaee M Wong LP. Understanding COVID-19 vaccine demand and hesitancy: a nationwide online survey in China. PloS Neglect Trop D. (2020) 14:e0008961. 10.1371/journal.pntd.0008961 33332359 PMC7775119

[13] Wu J Ma M Miao Y Ye B Li Q Tarimo CS. COVID-19 vaccination acceptance among Chinese population and its implications for the pandemic: a national cross-sectional study. Front Pub Health. (2022) 10:796467. 10.3389/fpubh.2022.796467 35211440 PMC8860971

[14] Champion V Skinner C. The health belief model. In: Glanz K Rimer B Viswanath K, editors. Health Behavior and Health Education: Theory, Research, and Practice (4th edition). San Francisco, CA: Jossey-Bass (2008). p. 45–65.

[15] Rosenstock I. What research in motivation suggests for public health. Am J Public Health Nations Health. (1960) 50:295–302. 10.2105/ajph.50.3_pt_1.295 14439041 PMC1373163

[16] Rosenstock I. Historical origins of the health belief model. Health Educ Monographs. (1974) 2:328–35. 10.1177/10901981740020040312859788

[17] Rosenstock I Strecher V Becker M. Social learning theory and the health belief model. Health Educ Q. (1988) 15:175–83. 10.1177/109019818801500203 3378902

[18] Carpenter C. A meta-analysis of the effectiveness of health belief model variables in predicting behavior. Health Commun. (2010) 25:661–9. 10.1080/10410236.2010.521906 21153982

[19] Chen H Li X Gao J Liu X Mao Y Wang R. Health belief model perspective on the control of COVID-19 vaccine hesitancy and the promotion of vaccination in China: web-based cross-sectional study. J Med Internet Res. (2021) 23:e29329. 10.2196/29329 34280115 PMC8425399

[20] Jiang T Zhou X Wang H Dong S Wang M Akezhuoli H. COVID-19 vaccination intention and influencing factors among different occupational risk groups: a cross-sectional study. Hum Vacc Immunother. (2021) 17:3433–40. 10.1080/21645515.2021.1930473 34289316 PMC8437552

[21] Tao L Wang R Han N Liu J Yuan C Deng L. Acceptance of a COVID-19 vaccine and associated factors among pregnant women in China: a multi-center cross-sectional study based on health belief model. Hum Vacc Immunother. (2021) 17:2378–88. 10.1080/21645515.2021.1892432 33989109 PMC8475603

[22] Yu Y Lau J She R Chen X Li L Li L. Prevalence and associated factors of intention of COVID-19 vaccination among healthcare workers in China: application of the Health Belief Model. Hum Vacc Immunother. (2021) 17:2894–902. 10.1080/21645515.2021.1909327 33877955 PMC8381834

[23] Wong M Wong E Huang J Cheung A Law K Chong M. Acceptance of the COVID-19 vaccine based on the health belief model: a population-based survey in Hong Kong. Vaccine. (2021) 39:1148–56. 10.1016/j.vaccine.2020.12.083 33461834 PMC7832076

[24] Tao L Wang R Liu J. Comparison of vaccine acceptance between COVID-19 and seasonal influenza among women in China: a national online survey based on health belief model. Front Med. (2021) 8:679520. 10.3389/fmed.2021.679520 34150811 PMC8211886

[25] Fishbein M Yzer M. Using theory to design effective health behavior interventions. Commun Theory. (2003) 13:164–83. 10.1111/j.1468-2885.2003.tb00287.x

[26] Hornik R Woolf K. Using cross-sectional surveys to plan message strategies. Soc Mark Q. (1999) 5:34–41. 10.1080/15245004.1999.9961044

[27] Corbin J Strauss A. Basics of Qualitative Research: Techniques and Procedures for Developing Grounded Theory (2nd edition.). Thousand Oaks, CA: Sage (2014).

[28] Braun V Clarke V. Using thematic analysis in psychology. Qual Res Psychol. (2006) 3:77–101. 10.1191/1478088706qp063oa

[29] Lincoln Y Guba E. Naturalistic Inquiry. Beverly Hills, CA: Sage. (1985).

[30] Brewer N Fazekas K. Predictors of HPV vaccine acceptability: a theory-informed, systematic review. Prev Med. (2007) 45:107–14. 10.1016/j.ypmed.2007.05.013 17628649

[31] Schmid P Rauber D Betsch C Lidolt G Denker M-L. Barriers of influenza vaccination intention and behavior: a systematic review of influenza vaccine hesitancy, 2005–2016. PLoS ONE. (2017) 12:e0170550. 10.1371/journal.pone.0170550 28125629 PMC5268454

[32] Sun Y Zhao Y Xue S Chen J. The theory development of traditional Chinese medicine constitution: a review. J Tradit Chin Med Sci. (2018) 5:16–28. 10.1016/j.jtcms.2018.02.007

[33] Green J Thorogood N. Qualitative Methods for Health Research. London: Sage (2004).

[34] Gong Z Tang Z Li J. What strategy is better for promoting COVID-19 vaccination? A comparison between gain-framed, loss-framed, and altruistic messages. Ann Behav Med. (2021) 56:325–31. 10.1093/abm/kaab070 34398184

[35] Yu Y Luo S Mo P Wang S Zhao J Zhang G. Prosociality and social responsibility were associated with intention of COVID-19 vaccination among university students in China. Int J Health Policy Manag. (2021) 2021:1–8. 10.34172/IJHPM.2021.64 34273931 PMC9808345

[36] Earley P. Social loafing and collectivism: a comparison of the United States and the people's republic of China. Adm Sci Q. (1989) 34:565–81. 10.2307/2393567

[37] Böhm R Betsch C Korn L Holtmann C. Exploring and promoting prosocial vaccination: a cross-cultural experiment on vaccination of health care personnel. BioMed Res Int. (2016) 2016:6870984. 10.1155/2016/6870984 27725940 PMC5048021

[38] Liao Q Cowling B Lam W Ng D Fielding R. Anxiety, worry and cognitive risk estimate in relation to protective behaviors during the 2009 influenza A/H1N1 pandemic in Hong Kong: ten cross-sectional surveys. BMC Infect Dis. (2014) 14:169. 10.1186/1471-2334-14-169 24674239 PMC3986671

[39] Triandis H. Individualism-collectivism and personality. J Pers. (2001) 69:907–24. 10.1111/1467-6494.696169 11767823

[40] Wang G Chen Y-N. Collectivism, relations, and Chinese communication. Chin J Commun. (2010) 3:1–9. 10.1080/17544750903528708

[41] Yue X Ng S. Filial obligations and expectations in China: current views from young and old people in Beijing. Asian J Soc Psychol. (1999) 2:21–6. 10.1111/1467-839X.00035

[42] Dutta M. Communicating about culture and health: theorizing culture-centered and cultural sensitivity approaches. Commun Theory. (2007) 17:304–28. 10.1111/j.1468-2885.2007.00297.x

[43] National Health Commission. Questions and Answers About COVID-19 Vaccination. (2021). Available online at: http://www.nhc.gov.cn/xcs/nwwd/202104/696521e535454821b63381c9f028a57a.shtml (accessed November 3, 2021).

[44] People's Daily. Twenty Questions to Understand the Key Points of COVID-19 Vaccination (2021). Available online at: https://weibo.com/2803301701/JBzwux7Jp?from=page_1002062803301701_profile&wvr=6&mod=weibotime&type=comment#_rnd1634302982741 (accessed November 3, 2021).

[45] Rogers E. Diffusion of preventive innovations. Addict Behav. (2002) 27:989–93. 10.1016/S0306-4603(02)00300-3 12369480

[46] Chen T Dai M Xia S. Perceived facilitators and barriers to intentions of receiving the COVID-19 vaccines among elderly Chinese adults. Vaccine. (2022) 40:100–6. 10.1016/j.vaccine.2021.11.039 34839994 PMC8604039

[47] Karabela S Coskun F Hosgor H. Investigation of the relationships between perceived causes of COVID-19, attitudes towards vaccine and level of trust in information sources from the perspective of Infodemic: the case of Turkey. BMC Pub Health. (2021) 21:1195. 10.1186/s12889-021-11262-1 34158015 PMC8219470

[48] Liu P Zhao X Wan B. COVID-19 information exposure and vaccine hesitancy: the influence of trust in government and vaccine confidence. Psychol Health Medi. (2021) 1–10. 10.1080/13548506.2021.2014910. [Epub ahead of print]. 34875950

[49] Moola S Gudi N Nambiar D Dumka N Ahmed T Sonawane I. A rapid review of evidence on the determinants of and strategies for COVID-19 vaccine acceptance in low—and middle-income countries. J Glob Health. (2021) 11:05027. 10.7189/jogh.11.05027 34912550 PMC8645216

[50] Paredes M Apaolaza V Marcos A Hartmann P. Predicting COVID-19 vaccination intention: the roles of institutional trust, perceived vaccine safety, and interdependent self-construal. Health Commun. (2021) 1–12. 10.1080/10410236.2021.1996685. [Epub ahead of print]. 34732090

[51] Yasuhara N Okamoto S Hamada M Uehara K Obana N Imamura T. Evaluation of Japanese people's perception of risk information for making decisions to receive influenza and rubella vaccinations. Health Expect. (2021) 24:2013–22. 10.1111/hex.13342 34432935 PMC8628585

[52] Kim S Pjesivac I Jin Y. Effects of message framing on influenza vaccination: understanding the role of risk disclosure, perceived vaccine efficacy, and felt ambivalence. Health Commun. (2019) 34:21–30. 10.1080/10410236.2017.1384353 29053369

[53] Peng L Guo Y Hu D. Information framing effect on public's intention to receive the COVID-19 vaccination in China. Vaccines. (2021) 9:995. 10.3390/vaccines9090995 34579232 PMC8471194

[54] Resnicow K Braithwaite R Dilorio C Glanz K. Applying theory to culturally diverse and unique populations. In: Glanz K Rimer B Lewis F, editors. Health Behavior and Health Education: Theory, Research, and Practice (3rd edition). San Francisco, CA: Jossey-Bass (2002). p. 485–509.

[55] Singhal A Rogers E. Combating AIDS: Communication Strategies in Action. London: Sage. (2003).

